# Eye-sidedness does not drive differences in growth and maturation in the Indian halibut (*Psettodes erumei*) from the Western Arabian Gulf

**DOI:** 10.1038/s41598-025-30930-5

**Published:** 2026-01-17

**Authors:** Yu-Jia Lin

**Affiliations:** 1 Institute of Marine Ecology and Conservation, National Sun Yat-sen University,, Kaohsiung , 804201 Taiwan; 2 Marine Science Oriented Ocean Technology Implementation Center, National Sun Yat-sen University, Kaohsiung , 80424 Taiwan

**Keywords:** Eye-sidedness, Psettodes, Life history traits, Polymorphic, Flatfish, Arabian gulf, Ecology, Ecology, Evolution, Zoology

## Abstract

The Indian halibut *Psettodes erumei* (Family Psettodidae), a primitive lineage of flatfishes exhibiting both sinistral and dextral morphs, provides a unique model for examining the evolutionary significance of morphological asymmetry in flatfishes. This study tested whether eye-sidedness influences somatic growth, body shape, and reproductive traits in *P. erumei* from the western Arabian Gulf. A total of 215 individuals were collected between 2020 and 2022, with sinistral and dextral morphs occurring in near-equal proportions. Model selection based on Akaike weights revealed that sex, rather than eye-sidedness, was the primary factor influencing length–weight relationships and growth, with females attaining significantly larger asymptotic lengths. Eye-sidedness had no detectable effect on gonadosomatic index patterns or length at 50% maturity. While Procrustes regression detected statistically significant differences in body landmarks and semilandmarks between morphs, these differences were minor and likely biologically negligible. Overall, the results support the hypothesis that eye-sidedness in *P. erumei* does not confer a measurable evolutionary advantage and imply that differences in developmental mechanisms, rather than adaptive advantages, played a primary role in fixing directional asymmetry in most flatfish lineages. Future research into the molecular and developmental pathways governing eye-sidedness will be essential for understanding why the vast majority of flatfish species exhibit monomorphic asymmetry.

**Supplementary Information:**

The online version contains supplementary material available at 10.1038/s41598-025-30930-5.

## Introduction

The most distinctive feature of adult flatfishes (Order Pleuronectiformes, Teleostei) is their pronounced cranial asymmetry, with both eyes positioned on one side of the head—a trait that emerges during the transition from a pelagic to a benthic lifestyle^1,2^. At hatching, flatfish larvae are bilaterally symmetrical. Metamorphosis into the juvenile stage involves the migration of one eye to the opposite side of the head, accompanied by a unidirectional reorganization of several anatomical structures^3^. This transformation typically results in lateral monomorphism, whereby all individuals of a species exhibit asymmetry on the same side. Most families are predominantly right-eyed (sinistral) or left-eyed (dextral)^4^. Accordingly, species and individuals are classified as sinistral or dextral based on the final position of the eyes.

However, an intriguing question arises that why some flatfish species are sinistral while others are dextral^2^. Phylogenetic relationships within Pleuronectiformes do not consistently correspond with eye-sidedness, and thus cannot fully explain this trait^5,6^. Notable exceptions include three species in the family Psettodidae and two in the genus *Platichthys*, the European flounder (*P. flesus*) and the starry flounder (*P. stellatus*), in which both sinistral and dextral individuals were observed with varying proportions^2,7,8^. As a result, *P. flesus* and *P. stellatus* have been widely used as model species for investigating morphological, ecological, and behavioral differences between the two morphs^7–11^.

The family Psettodidae represents the most basal lineage of extant flatfishes, comprising a single genus, *Psettodes*, with three recognized species: the spottail spiny turbot (*Psettodes belcheri*), the spiny turbot (*P. bennetti*), and the Indian halibut (*P. erumei*)^12^. As a plesiomorphic group, Psettodidae exhibits indeterminate asymmetry, with sinistral and dextral individuals occurring at random with approximately equal frequencies^4,13^. In contrast, most other lineages within Pleuronectiformes exhibit fixed directional asymmetry, with either sinistral or dextral forms predominating^4^. The extinct genus *†Amphistium*, one of the earliest known pleuronectiforms, also exhibited indeterminate ocular asymmetry. Thus, indeterminate orbital migration is considered the ancestral state in flatfishes, while fixed eye-sidedness represents a derived state^1,14^.

Somatic growth and maturation patterns are critical life-history traits, with larger body sizes generally associated with larger fecundity nonlinearly^15^ and better evolutionary fitness^16^. Variation in growth trajectories can also contribute to fluctuations in population size^17^. Somatic growth is typically characterized by the relationship between body weight and age^18^. Length-weight relationships are widely used to estimate body condition, under the assumption that heavier individuals of a given length are in better physiological condition^19^. The von Bertalanffy growth model, a widely applied bioenergetic model, describes the relationship between body length and age and is the most commonly used framework for modelling fish growth^20^.

*Psettodes erumei* is a moderately sized flatfish widely distributed across tropical and subtropical waters of the Indian Ocean and western Pacific^12^. It is distinguished by well-defined myotomes, prominent canine teeth, and an approximately equal occurrence of sinistral and dextral individuals^13,21^. In adults, the upper body surface is grey-brown, providing effective camouflage on muddy sand substrates, while juveniles exhibit vertical stripes on the dorsal surface. Due to its relatively large size, *P. erumei* is frequently targeted by fisheries throughout its geographic range^21–24^.

Although sinistral and dextral individuals within the same flatfish species are not exact mirror images of one another, and various morphological, ecological, and behavioral differences have been reported^7–11^, it remains unclear whether such differences are directional and cumulative, potentially resulting in divergent somatic growth trajectories, with implications for evolutionary fitness^15,16^, and population size fluctuations^17^.

This study aims to test whether sinistral and dextral individuals differ in life-history traits, as assessed through length–weight relationships, von Bertalanffy growth, and maturity parameters, using *Psettodes erumei* as a model species. Specifically, we address three objectives: (1) to determine whether sinistral and dextral individuals are randomly distributed, (2) to assess whether sinistral and dextral individuals are mirror-images of each other, and (3) to compare length–weight relationships, growth, and maturation parameters between sexes and between sinistral and dextral individuals.

## Results

### Sample collection

A total of 215 *Psettodes erumei* individuals were collected from the western Arabian Gulf between 2020 and 2022, comprising 109 females, 96 males, and 10 sexually undifferentiated specimens. Total length ranged from 132 to 652 mm, total weight from 26 to 4360 g, and age from 0 to 15 years (Table 1). The sample included 107 dextral and 108 sinistral individuals, a distribution not significantly different from random (χ^2^ test, $\:{{\upchi\:}}_{1}^{2}$ = 0.005, *p* = 0.946). Eye-sidedness showed no significant association with sex, either ($\:{{\upchi\:}}_{2}^{2}$ = 3.086, *p* = 0.214).

**Table 1:** Sample size for somatic measurement (N) and ageing (N_Age_), and mean (± SD, range in the parenthesis) total length (L_T_, mm), total somatic weight (W_T_, g), and age (years) of *Psettodes erumei* by eye-sidedness (dextral or sinistral) and sexes (F = female, M = male, and U = undifferentiated individuals) collected from the Western Arabian Gulf from 2020 to 2022.

Eye sidedness	Sex	*N*	*N*_Age_	L_T_	W_T_	Age
Dextral	F	58	55	460 ± 92 (309 ~ 652)	1616 ± 989 (385 ~ 4360)	3.4 ± 3.0 (0.5 ~ 15.0)
	M	43	41	386 ± 57 (276 ~ 473)	897 ± 401 (280 ~ 1624)	2.1 ± 1.3 (0.5 ~ 6.0)
	U	7	7	276 ± 70 (132 ~ 352)	328 ± 173 (26 ~ 594)	0.9 ± 0.6 (0.0 ~ 2.0)
Sinistral	F	51	45	453 ± 95 (280 ~ 632)	1562 ± 981 (267 ~ 3960)	3.2 ± 2.6 (0.5 ~ 13.0)
	M	53	46	381 ± 58 (269 ~ 471)	860 ± 386 (238 ~ 1570)	2.1 ± 1.2 (0.5 ~ 6.0)
	U	3	3	229 ± 84 (132 ~ 284)	212 ± 162 (26 ~ 309)	0.7 ± 0.6 (0.0 ~ 1.0)

The sex-specific length-weight relationship was best supported by the data, exhibiting the lowest AIC and strong support (W_AIC_ = 0.886), whereas eye-sidedness and full model had minimal effect on this relationship with very low support from the data (δAIC = 8.5 and 9.5 and W_AIC_ = 0.013 and 0.008, respectively, Table 2). The exponent (*b*) of the length-weight relationship of the males was slightly higher than that of the females (Table 3).

**Table 2:** Number of parameters (N_P_), values of Akaike information criterion (AIC), AIC difference (δAIC), and AIC weight (W_AIC_) of somatic length weight relationship (LW-relationship), von Bertalanffy growth curves and maturation curves with different combinations of eye-sidedness and sex effect. The best model with the lowest AIC value was in bold. Csize is the centroid size in the landmark analysis, which is used to account for allometric size effect. The maturation model with the eye sidedness as a covariate was not converged so that it not included in the model comparison.

Process	Effect	*N*_*P*_	AIC	δAIC	W_AIC_
LW relationship	Null	3	−1057.2	4.5	0.093
	Eye sidedness	5	−1053.2	8.5	0.013
	**Sex**	**5**	−1061.7	**0.0**	**0.886**
	Eye sidedness, Sex	9	−1052.2	9.5	0.008
Procrustes regression on	Null	1	−981.0	5.0	0.034
landmarks and semilandmarks	**log(Csize)**	**2**	**-986.0**	**0.0**	**0.395**
	Eye sidedness, log(Csize)	3	−984.6	1.4	0.199
	Sex, log(Csize)	3	−985.0	1.0	0.248
	Eye sidedness, Sex, log(Csize)	4	−983.6	2.4	0.124
von Bertalanffy growth model	Null	4	1573.9	57.9	0.000
	Eye sidedness	7	1578.0	62.0	0.000
	**Sex**	**7**	**1516.0**	**0.0**	**1.000**
	Eye sidedness, Sex	13	1567.4	51.4	0.000
Maturation	**Total length**	**2**	**60.3**	**0.0**	**0.822**
	Total length, Sex	4	63.4	3.1	0.178
	Total length, Sex, Eye sidedness	Not converged			

Bold values indicate the best model with the smallest AIC value (equivalent to δAIC = 0).

**Table 3:** Parameter estimates (± standard error) in the allometric total length (L_T_) - total weight (W_T_) relationship: $\:{W}_{T}=a{L}_{T}^{b}$, observed maximum total length and age (L_max_ and A_max_), von Bertalanffy growth model, and logistic maturation model of *Psettodes erumei* by sex.

Relationship	Parameter	Female	Male
Maximum observed total length	L_max_ (mm)	652	473
Maximum observed age	A_max_ (years)	15	6
Somatic length-weight relationship	ln(a)	−12.251 ± 0.144	−12.560 ± 0.175
	b	3.182 ± 0.024	3.237 ± 0.030
von Bertalanffy growth model	*L*_∞_ (mm)	594 ± 14	448 ± 8
	*K* (year^− 1^)	0.429 ± 0.048	0.982 ± 0.112
	*L*_0_ (mm)	197 ± 17	155 ± 20
	*t*_0_ (years)	-0.971 ± 0.177	−0.434 ± 0.105
Logistic maturation model	*L*_*50%*_ (mm)	288 ± 7	
		296 ± 9	282 ± 12

## Landmark analysis

**Fig. 1:**
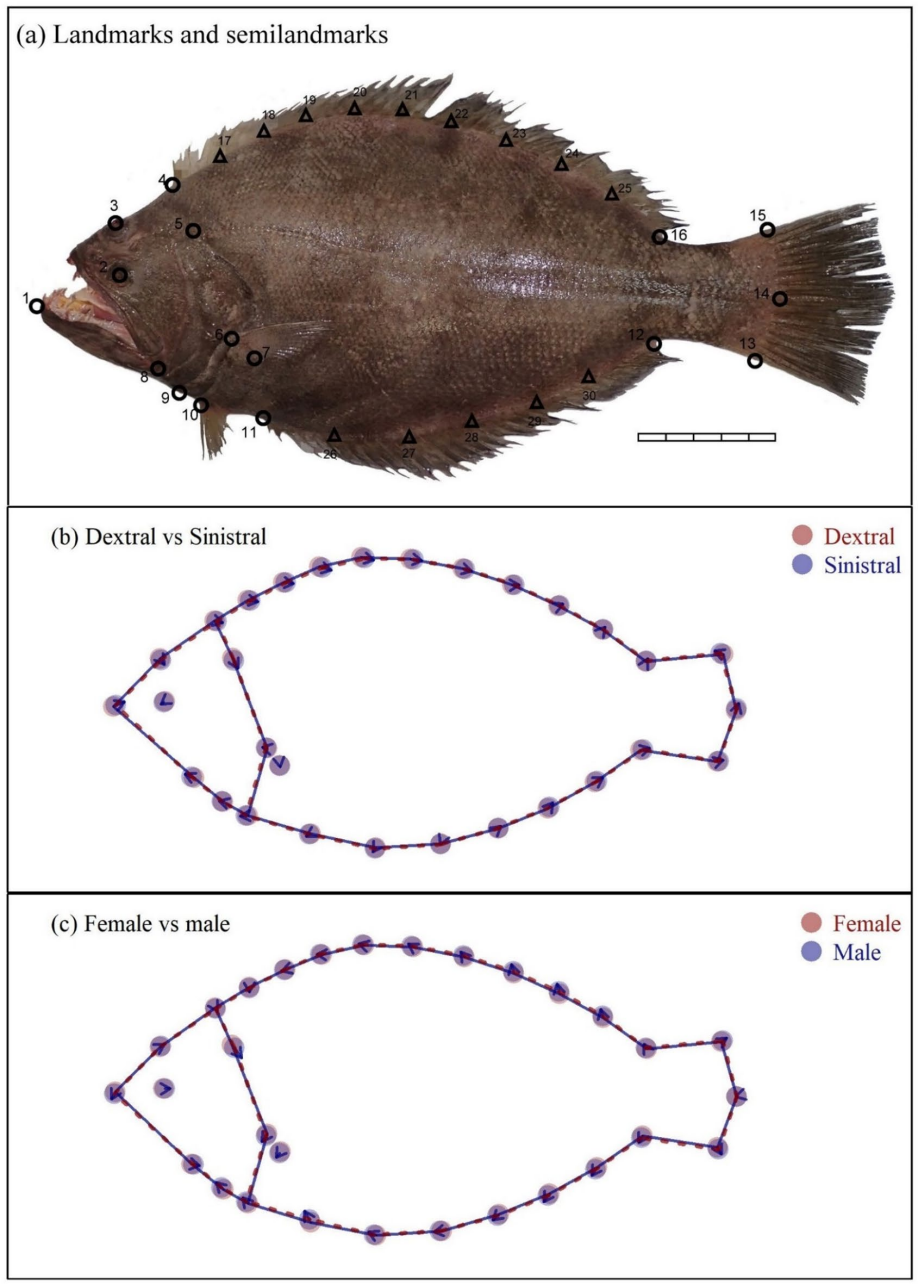
(**a**) Landmarks (open circles), semilandmarks (open triangles) on *Psettodes erumei*. The landmarks included: (1) tip of the lower jaw, (2 and 3) center of the eyes, (4) anterior insertion of the dorsal fin, (5) beginning of the lateral line, (6 and 7) superior and inferior insertion of the pectoral fin, (8) posterior extremity of premaxillar, (9) insertion of the operculum on the lateral profile, (10) insertion of first ray of the pelvic fin, (11) anterior insertion of the anal fin, (12) posterior insertion of the anal fin, (13 and 15) dorsal and ventral extremes of the caudal fin insertion, (14) posterior extremity of the caudal peduncle, (16) posterior insertion of the dorsal fin. (17–25): semilandmarks collected on the dorsal profile, and (26–30) semilandmarks collected on the ventral profile. Scale bar = 5 cm. Procrustes superimposition showing the pair-wise differences in shapes between (**b**) dextral (red broken lines and dots) and sinistral (blue solid lines and dots) individuals and (**c**) females (red broken lines and dots) and males (blue solid lines and dots). Arrows indicate the difference vector, which was amplified ten times for clarity.

Sinistral individuals exhibited greater morphological difference to dextral individuals in the position of the lateral line origin (landmark #5), the dimensions of the caudal peduncle (landmarks #13–15), and the anterior dorsal curvature (semilandmarks #17–23; Fig. 1b). Relative to females, males showed more pronounced variation in the lateral line origin, caudal peduncle size, and ventral curvature (semilandmarks #17–23; Fig. 1c). The landmarks and semilandmarks were significantly different between sex (Procrustes regression with 9999 permutations, *p* = 0.0158) and eye-sidedness (*p* = 0.0152). Nonetheless, these differences were minor, and the overall body outlines derived from landmarks and semilandmarks largely overlapped between sinistral and dextral individuals (Fig. 1b), as well as between sexes (Fig. 1c). Among the Procrustes regression models, the one incorporating only allometric length effects had the lowest AIC value and the strongest support (W_AIC_ = 0.395), whereas models accounting for sex, eye-sidedness, or both were less supported (W_AIC_ = 0.248, 0.199, and 0.124, respectively; Table 2). These results suggest that sex and eye-sidedness exert limited influence on overall body shape.

## Growth model

**Fig. 2:**
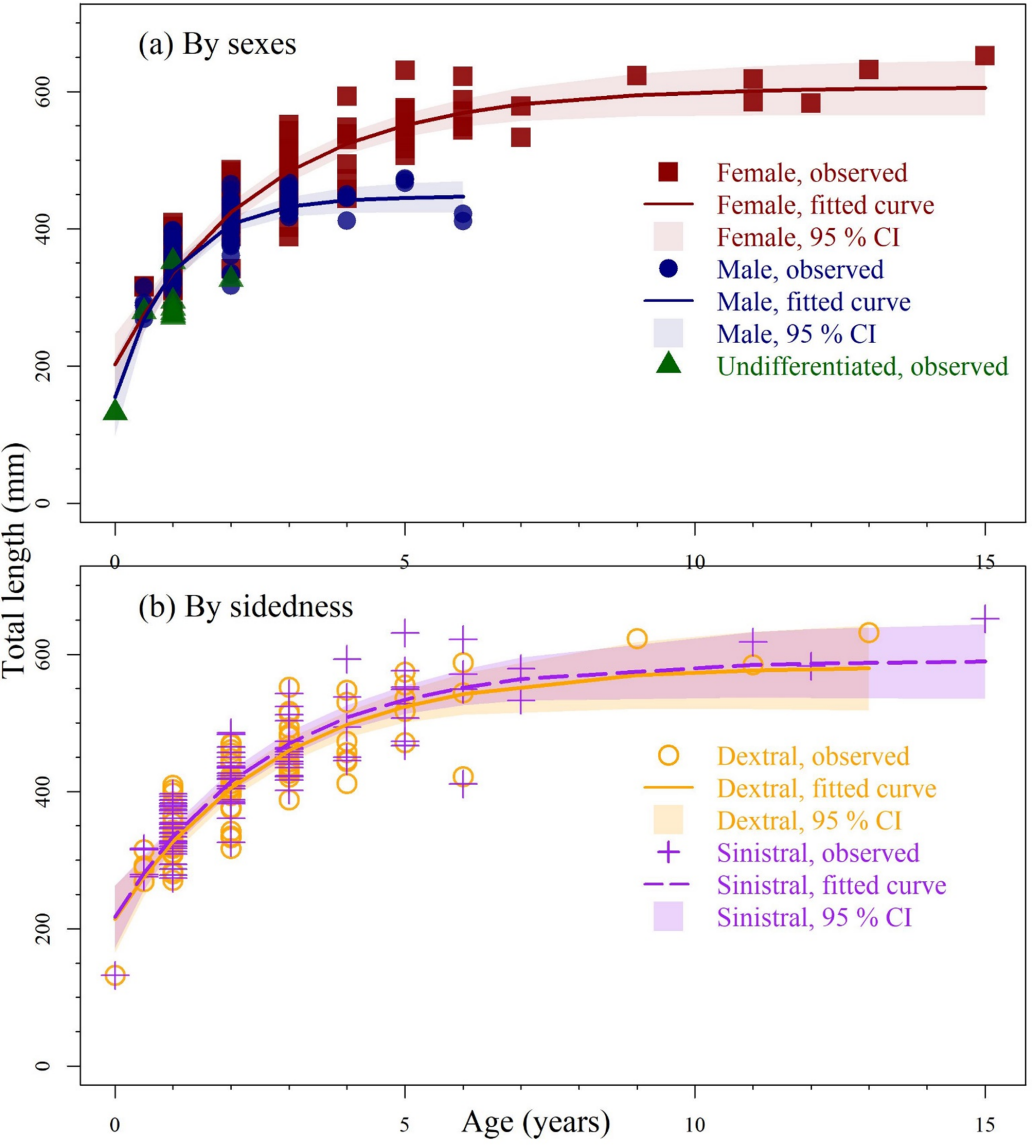
Observed length-at-ages (symbols), fitted von Bertalanffy growth curves (lines), and corresponding 95% confidence interval of fitted line (shaded area) (**a**) by sexes (red = females, blue = males, and green = undifferentiated) and (**b**) by eye-sidedness (yellow solid line = dextral and purple broken line = sinistral).

The von Bertalanffy growth curves differed markedly between sexes, with non-overlapping 95% confidence intervals after age 3 (Fig. 2a). In contrast, eye-sidedness had no discernible effect on growth, as the curves for sinistral and dextral individuals were generally close to each other with overlapped 95% confidence intervals (Fig. 2b). The sex-specific growth model was best supported by the length-at-age data, with a dominant support (WAIC ≈ 1; Table 2), whereas eye-sidedness had minimal impact and negligible support (WAIC ≈ 0; Table 2). Females exhibited a larger asymptotic length (L_∞_ = 594 mm) and a lower growth coefficient (K = 0.429 year⁻¹) compared to males (L_∞_ = 448 mm; K = 0.982 year⁻¹; Table 3).

## Maturation model and seasonal GSI pattern

**Fig. 3:**
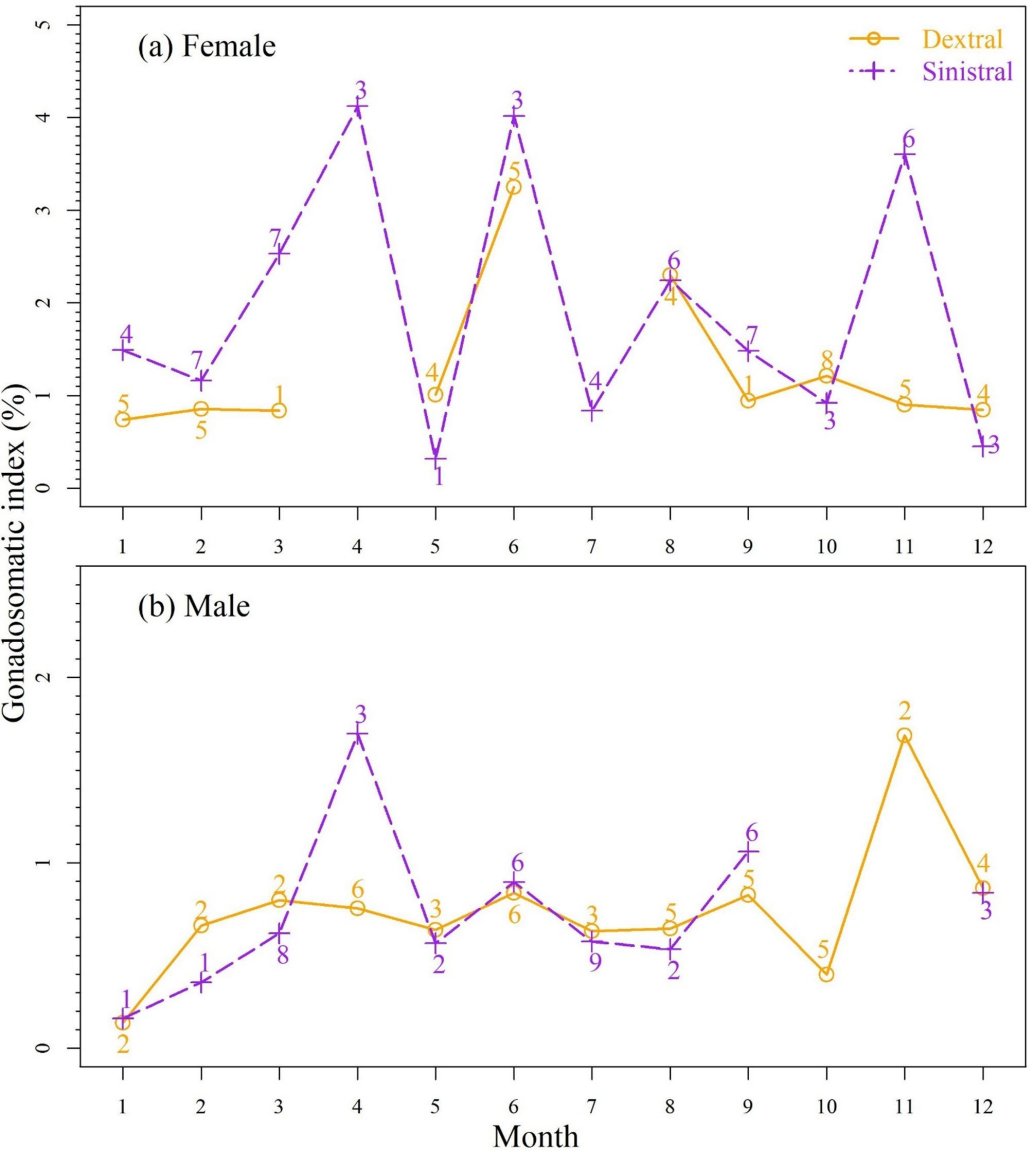
Monthly mean gonadosomatic index of (**a**) females and (**b**) males by eye-sidedness (yellow solid line = dextral and purple broken line = sinistral). Numbers indicate the sample size.

The logistic maturation model with eye-sidedness did not converge, possibly due to limited sample size, and was thus excluded from model comparison. The length-only model that the maturation at length did not differ between females and males was best supported by the maturation data with strong support (WAIC = 0.822, Table 2). The estimate (± standard error) of the sex-pooled length at 50% maturity was 288 ± 7 mm (Table 3). Females exhibited peak gonadosomatic index (GSI) values in April, June, and November, whereas males showed prominent peaks in April and November. Despite limited sample sizes, monthly GSI patterns were generally consistent between sinistral and dextral individuals (Fig. 3).

## Discussion

By calculating Akaike weights within an information-theoretic framework, it is possible to quantify the relative strength of evidence supporting alternative models^25^. For *Psettodes erumei* from the western Arabian Gulf, eye-sidedness appears to have no meaningful effect on length-weight relationships, von Bertalanffy growth parameters, or logistic maturation models. This is supported by high Akaike weights (> 0.8) favoring models without eye-sidedness, as well as overlapping confidence intervals (Fig. 2b). Similarly, the generally overlapping monthly gonadosomatic index (GSI) patterns between sinistral and dextral individuals (Fig. 3) suggest possibly similar reproductive seasons across morphs.

Eye-sidedness may exert a minor yet statistically significant effect on body shape, indicating that sinistral and dextral individuals are not perfect mirror images of each other. In polymorphic flounders such as *Platichthys flesus* and *Platichthys stellatus*, eye-sidedness is associated with significant morphological differences, which have been linked to differences in foraging behavior^7^, feeding preference^8,10^, and swimming performance^11^. It is therefore plausible that *Psettodes erumei* may also exhibit similar behavioral or ecological differences between morphs, which could be explored in future studies, e.g.^26^

However, subtle morphological differences observed in *Psettodes erumei* between morphs may have limited biological relevance^27^, as landmarks and semilandmarks largely overlapped between sinistral and dextral individuals (Fig. 1b). Moreover, even if *Psettodes erumei* displays variation in feeding behavior or dietary preferences between morphs, insignificant differences between the length-weight relationship and von Bertalanffy growth curve (Table 2; Fig. 2) indicated that such differences are likely to be biologically minor, non-directional, and insufficiently cumulative to produce detectable variation in somatic growth relationships.

Sinistral and dextral *Psettodes erumei* individuals from the western Arabian Gulf were randomly distributed without apparent influence of sex. This finding confirms previous studies suggesting that both morphs are distributed randomly within populations^4^. According to the competitive exclusion principle, morphs competing for the same limiting resources cannot stably coexist, as even a slight advantage of one form would eventually lead to the exclusion of the other^28^. Therefore, the observed equal proportions of sinistral and dextral individuals suggest either the existence of mechanisms that reduce intraspecific competition, such as niche differentiation or temporal segregation^29,30^, or the absence of a competitive advantage associated with eye-sidedness for *Psettodes erumei*. Overlapping monthly gonadosomatic index (GSI) patterns between morphs rule out temporal separation in reproductive season^31^, and the absence of significant differences in somatic growth relationships supports the hypothesis that eye-sidedness does not confer a measurable competitive advantage.

**Fig. 4:**
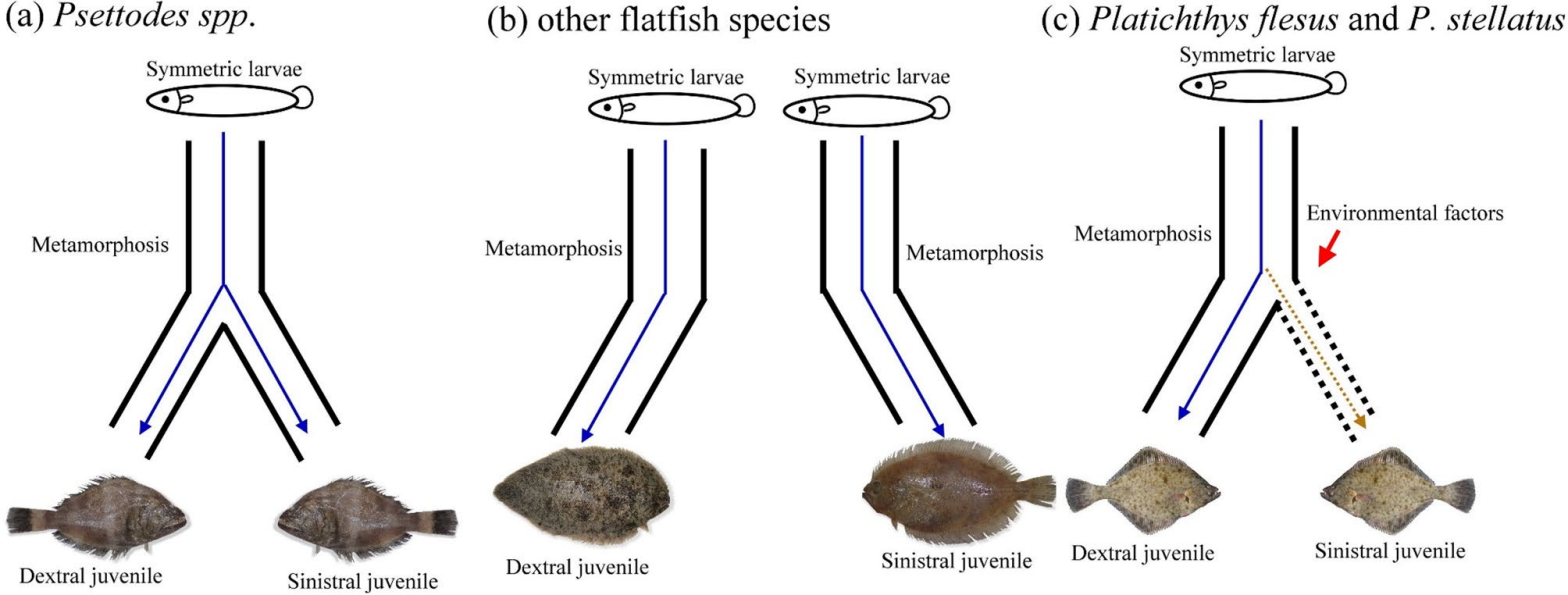
Three patterns of eye-sidedness: (**a**) *Psettodes spp.*, where the eye-sidedness is randomly distributed, (**b**) other flatfish species, where the eye-sidedness is fixed, and (**c**) in rare cases (such as *Platichthys flesus* and *P. stellatus*), where the eye-sidedness varies among studies and is affected by environmental factors.

In contrast to *Psettodes* spp., where eye-sidedness is randomly distributed (Fig. 4a), and most other flatfish species, where eye-sidedness is fixed (Fig. 4b), *Platichthys flesus* and *P. stellatus* exhibit variable sidedness, ranging from predominantly dextral to completely sinistral (Fig. 4c;^9^. The proportions of eye-sidedness are heritable, with offspring from dextral × dextral crosses being predominantly dextral, and vice versa^5,32^. Moreover, these proportions vary geographically^5,9,33^, suggesting environmental influences, such as salinity^33^, also play a role in determining sidedness^32^. These differences in eye-sidedness are associated with ecological, behavioural, and dietary divergence, e.g^9,10^. Accordingly, the present study *on Psettodes erumei* serves as a demonstration that examining key life-history traits, such as age, growth, and maturation parameters, can help assess whether observed morphological variation carries evolutionary significance.

*P. erumei* from the western Arabian Gulf exhibited marked sexual dimorphism in size and growth, with females displaying a distinct growth trajectory and attaining larger sizes than males. This pattern has similarly been observed in *P. erumei* populations from the western Indian Ocean^21^; the Bay of Bengal^34^ and western Australia^23^, as well as in other flatfish species such as the Greenland halibut (*Reinhardtius hippoglossoides*^35^, , Atlantic halibut (*Hippoglossus hippoglossus*^36^, , and olive flounder (*Paralichthys olivaceus*^37^, . The larger size of females is likely driven by fecundity selection, as reproductive output increases nonlinearly with body size until reaching an evolutionary trade-off point with mortality^15,38^. Conversely, the smaller size of males suggests limited male-male competition and low levels of sperm competition^38^.

The estimated length at 50% maturity (L_50%_) for *P. erumei* in the western Arabian Gulf was 296 mm (95% CI: 277–314) for females and 282 mm (95% CI: 258–305) for males, closely aligning with values reported for males from western Australia (268 mm, 95% CI: 250–288)^23^. However, substantially larger L_50%_ values were obtained for *P. erumei* from southern India four decades ago (371–390 mm)^39^ and from the entrance of the Arabian Gulf to the Oman Sea in 2016 (Hormozgan, Iran, 382 mm)^24^, which fell outside the 95% confidence interval for females in the present study, suggesting a statistically significant difference. This spatial and temporal variation in L_50%_ may be attributed to multiple factors, including long-term fishing pressure^39^ potentially driving evolutionary shifts towards earlier maturation at smaller sizes^40,41^, geographical variation in life history traits^42,43^, and possible genetic divergence between populations from the western Arabian Gulf and southern India, as observed in other flatfish species^44^. Therefore, this study provides essential baseline information for future comparative studies.

In conclusion, this study demonstrates that eye-sidedness in *Psettodes erumei* from the western Arabian Gulf is randomly distributed. While minor morphological differences exist between sinistral and dextral individuals, they are not sufficiently distinct to suggest functional divergence, as no significant differences in somatic growth, maturation, or reproductive timing. In contrast, sexual dimorphism strongly influences life-history traits, with females attaining larger asymptotic sizes. The absence of directional or cumulative effects of eye-sidedness on growth and reproduction suggests that asymmetry in *P. erumei* is evolutionarily neutral. Together with the lack of correspondence between phylogenetic relationships and eye-sidedness across Pleuronectiformes^5,6^, these findings imply that differences in developmental mechanisms^6,14^, rather than adaptive advantages, played a primary role in fixing directional asymmetry in most flatfish lineages. Future research into the molecular and developmental pathways governing eye-sidedness will be essential for understanding why the vast majority of flatfish species exhibit monomorphic asymmetry.

## Materials and methods

### Sample collection

This study did not conduct any experiments on live fish individuals and all experiments were performed in accordance with relevant guidelines and regulations. Specimens of *Psettodes erumei*, mostly caught by bottom gillnets, were collected irrespective of their eye-sidedness during weekly visits to local retail fish markets in Dammam and Jubail, Eastern Province, Saudi Arabia, from 2020 to 2022. The collection protocol of Lin et al.^45^ was followed to obtain specimens from the Arabian Gulf across the full size range, with emphasis on large individuals to reliably estimate asymptotic length^46^. In the laboratory, each specimen was photographed with the eye-side facing upwards, after which eye-sidedness, dextral or sinistral, was recorded. Somatic measurements included total length (L_T_) and standard length (L_S_) to the nearest mm, and total weight (W_T_) to the nearest g.

The gonads were extracted from each individual, weighed to the nearest 0.01 g, and examined macroscopically for the determination of the sex following Benzik et al.^47^. The gonadosomatic index (GSI) was calculated for each individual using the following formula: $\:GSI=100\times\:{W}_{G}\times\:{W}_{T}^{-1}$, where *W*_G_ is the gonad weight in g and *W*_T_ is the total somatic weight in g. Monthly mean gonadosomatic index values were calculated by sex and eye-sidedness to represent the seasonal maturation patterns.

## Landmark analysis

The difference in the body form between sinistral and dextral individuals was examined by the geometric morphometric method. Specimens exhibiting different eye-sidedness were examined following the protocol of Schilthuizen et al.^48^. To standardize orientation, images of sinistral *Psettodes erumei* individuals were horizontally mirrored using Adobe Photoshop, ensuring all specimens were analyzed in a consistent directional frame. Landmark configuration followed Russo et al.^8^, comprising 16 fixed landmarks and two sets of semilandmarks, nine and five equidistant points, placed along the anterior and posterior insertions of the dorsal and anal fins, respectively (Fig. 1a). Digitization of landmarks and semilandmarks was performed using the *geomorph* package^49^ in *R*^50^. Generalized Procrustes analysis (Claude, 2008) was employed to align specimens and minimize non-shape variation, quantified by Procrustes distance, in accordance with Lin et al.^51^. Shape variation attributable to covariates (eye-sidedness and sex) was assessed via Procrustes regression with 9,999 permutations. Allometric effects were accounted for using the log-transformed centroid size^52^. Morphological differences between groups were visualized by plotting mean landmark configurations, with displacement vectors indicating the direction and magnitude of shape change.

### Age, growth, and maturation

The sagittal otoliths were embedded, sectioned, polished, and photographed following the ageing procedure by Lin et al.^45,51^. The age was determined by counting the number of increments on the otolith transverse section (Appendix Fig. 2), whose annual periodicity has been validated by Das and Mishra^34^ and Coulson and Poad^23^. Age readings from Coulson and Poad^23^ were also used as supporting information in determining the locations of the first annulus and other annuli. Otoliths without the first annulus were regarded as 0.5-year-old, as Lin et al.^45^.

A re-parameterized von Bertalanffy growth function in which the age at length zero (t_0_) was replaced by the length at zero age (L_0_) was fitted using length-at-age data:

$$\:{L}_{t}={L}_{\infty\:}-{(L}_{\infty\:}-{L}_{0}){e}^{-K\times\:t}$$,

where *L*_∞_ is the asymptotic length, *K* is the von Bertalanffy growth coefficient, and *t* is the age. The model parameters were estimated using the maximum likelihood approach with a normal distribution for the conditional distribution of length at age given the model.

The maturation by length was modeled by the logistic model: $\:P\left({L}_{t}\right)=(1+{e}^{\alpha\:+\beta\:{L}_{t}}{)}^{-1}$, where *P*(*L*_*t*_) is the proportion of individuals mature at length *L*, *α* and *β* are the intercept and slope parameters, respectively. The parameters were estimated by maximum likelihood in the generalized linear model framework under a binomial distribution and logit link function. The length at 50% (*L*_50%_) maturity was calculated using the following: $\:{L}_{50\%}=-\widehat{\alpha\:}{\widehat{\beta\:}}^{-1}$, where $\:\widehat{\alpha\:}$ and $\:\widehat{\beta\:}$ is the estimate of the intercept and slope parameters, respectively. The standard error of *L*_50%_ and *L*_95%_ was calculated by the delta method using the R package *msm*^53^.

### Statistical analysis

The effect of sex on the proportion of sidedness was examined by χ^2^ test. Model selection approach based on the information theory^25^ was applied to select the best model with different combinations of sex and eye-sidedness effect for somatic length-weight relationship, Procrustes regression for the landmark and semilandmarks data, von Bertalanffy growth curve for the lengths-at-age data, and logistic maturation model for the maturation data. Candidate models with different combinations of effects were fitted, and corresponding Akaike Information Criterion (AIC) values were calculated: $\:\mathrm{A}\mathrm{I}\mathrm{C}\:=\:2{\mathrm{N}}_{p}\:-\:2\mathrm{l}\mathrm{n}\left(\widehat{L}\right)$, where N_p_ = number of model parameters and$\:\:\widehat{L}\:$is the maximized likelihood value. In case of the Procrustes regression, AIC value was calculated from the residual sum of squares (RSS), by assuming a normal distribution of the residuals: $\:\mathrm{A}\mathrm{I}\mathrm{C}\:=\:2{\mathrm{N}}_{p}+\:2\mathrm{l}\mathrm{n}\left(\frac{\mathrm{R}\mathrm{S}\mathrm{S}}{N-{N}_{p}}\right)-2C$, where N = total number of specimens, C is the normalizing constant.

The model with the lowest AIC value was selected as the best model supported by the data. AIC difference (δAIC), as the difference in AIC with respect to the AIC of the best candidate model, was calculated: AIC_i_ – min(AIC), where AIC_i_ was the AIC value for the candidate model i. Values of δAIC were regarded as a continuous measure of the strength of evidence that models with δAIC 2 were considered to be essentially as good as the best model, models with 4 δAIC 7 have considerably less support, and models with δAIC 10 were considered implausible^25^. Akaike weight (WAIC) for each model was calculated from δAIC: $$\:{W}_{AIC,i}=\raisebox{1ex}{${e}^{{-0.5\times\:\delta\:AIC}_{i}}$}\!\left/\:\!\raisebox{-1ex}{$\sum\:_{i}\left({e}^{-0.5\times\:{\delta\:AIC}_{i}}\right)$}\right.$$, as the relative weight of support (from 0 to 1) in favor of model i being the actual best model among the models tested^25^. All the computation was completed in *R*^50^, and the significance level α is set at 0.05.

## Supplementary Information

Below is the link to the electronic supplementary material.

Supplementary Material 1

## Data Availability

The data will be available upon reasonable request.

## References

[1] Friedman, M. The evolutionary origin of flatfish asymmetry. *Nature* **454** (7201), 209–212 (2008). 18615083 10.1038/nature07108

[2] Gibson, R. N., Nash, R. D., Geffen, A. J. & Van der Veer, H. W. (eds) *Flatfishes: Biology and Exploitation* (Wiley, 2015).

[3] Brewster, B. Eye migration and cranial development during flatfish metamorphosis: a reappraisal (Teleostei: Pleuronectiformes). *J. Fish Biol.* **31** (6), 805–833 (1987).

[4] Munroe, T. A. Systematic diversity of the Pleuronectiformes. Flatfishes: biology and exploitation. 13–51. (2014).

[5] Policansky, D. Flatfishes and the inheritance of asymmetries. *Behav. Brain Sci.* **5** (2), 262–265 (1982).

[6] Duarte-Ribeiro, E. et al. Phylogenomic and comparative genomic analyses support a single evolutionary origin of flatfish asymmetry. *Nat. Genet.* **56** (6), 1069–1072 (2024). 38802566 10.1038/s41588-024-01784-w

[7] Bergstrom, C. A. & Palmer, A. R. Which way to turn? Effect of direction of body asymmetry on turning and prey strike orientation in Starry flounder *Platichthys stellatus* (Pallas)(Pleuronectidae). *J. Fish Biol.* **71** (3), 737–748 (2007).

[8] Russo, T. et al. Right or wrong? Insights into the ecology of sidedness in European flounder, *Platichthys Flesus*. *J. Morphol.* **273** (3), 337–346 (2012). 22025394 10.1002/jmor.11027

[9] Bergstrom, C. A. Morphological evidence of correlational selection and ecological segregation between dextral and sinistral forms in a polymorphic flatfish, *Platichthys stellatus*. *J. Evol. Biol.* **20** (3), 1104–1114 (2007). 17465920 10.1111/j.1420-9101.2006.01290.x

[10] Bergstrom, C. A. & Reimchen, T. E. Isotopic trophic segregation associated with asymmetry direction in a polymorphic flatfish, platichthys stellatus (Pleuronectiformes: Pleuronectidae). *Biol. J. Linn. Soc.* **123** (4), 754–766 (2018).

[11] Bergstrom, C. A., Alba, J., Pacheco, J., Fritz, T. & Tamone, S. L. Polymorphism and multiple correlated characters: do flatfish asymmetry morphs also differ in swimming performance and metabolic rate? *Ecol. Evol.* **9** (8), 4772–4782 (2019). 31031943 10.1002/ece3.5080 PMC6476766

[12] Fricke, R., Eschmeyer, W. N. & Van der Laan, R. (eds). Eschmeyer’s catalog of fishes: genera, species, references. Electronic version accessed 22 Aug 2025. (2025). http://researcharchive.calacademy.org/research/ichthyology/catalog/fishcatmain.asp

[13] Das, M. & Mishra, B. Observation on the occurrence of dimorphic forms of *Psettodes erumei* (Bloch and Schn.), an Indian halibut. *J. Fisheries*. **36** (4), 337–338 (1989).

[14] Suzuki, T. & Tanaka, M. *Development and Regulation of External Asymmetry during Flatfish Metamorphosis*171–184 (Biology and exploitation, 2014).

[15] Barneche, D. R., Robertson, D. R., White, C. R. & Marshall, D. J. Fish reproductive-energy output increases disproportionately with body size. *Science* **360** (6389), 642–645 (2018). 29748282 10.1126/science.aao6868

[16] Stearns, S. C. Life history evolution: successes, limitations, and prospects. *Naturwissenschaften* **87** (11), 476–486 (2000). 11151666 10.1007/s001140050763

[17] Stawitz, C. C. & Essington, T. E. Somatic growth contributes to population variation in marine fishes. *J. Anim. Ecol.* **88** (2), 315–329 (2019). 30381829 10.1111/1365-2656.12921

[18] Hilborn, R. & Walters, C. J. (eds) *Quantitative Fisheries Stock Assessment: choice, Dynamics and Uncertainty* (Springer New York, 1992).

[19] Froese, R. Cube law, condition factor and weight–length relationships: history, meta-analysis and recommendations. *J. Appl. Ichthyol.* **22** (4), 241–253 (2006).

[20] Quinn, T. J. & Deriso, R. B. *Quantitative Fish Dynamics* (Oxford University Press, 1999).

[21] Darracott, A. Availability, morphometrics, feeding and breeding activity in a multi-species, demersal fish stock of the Western Indian ocean. *J. Fish Biol.* **10** (1), 1–16 (1977).

[22] Mathew, G., Feroz Khan, M. & Nandakumaran, K. *Present Status of Exploitation of Fish and Shellfish Resources: Flatfishes and Flatheads* Vol. 45, pp.197–204 (CMFRI Bulletin, 1992).

[23] Coulson, P. G. & Poad, J. A. Biological characteristics of the primitive flatfish Indian halibut (*Psettodes erumei*) from the tropical Northeastern Indian Ocean, including implications of the use of incorrect aging methods on mortality estimates. *Fish. Bull.* **119** (2–3), 168–183 (2021).

[24] Ghanbarzadeh, M., Kamrani, E., Ranjbar, M. S., Aalarpouri, A. & Walters, C. Reproductive biology of Indian halibut, *Psettodes erumei* from the Northern Persian Gulf and Oman sea (Teleoststei: Psettodidae). *Iran. J. Ichthyol.* **8** (1), 1–13 (2021).

[25] Burnham, K. P. & Anderson, D. R. *Model Selection and Multimodel Inference* (Springer, 2002).

[26] Ghanbarzadeh, M., Kamrani, E., Ranjbar, M. S., Salarpouri, A. & Walters, C. Food and feeding habits of Indian halibut, *Psettodes erumei* from the North of the Persian Gulf and Oman sea. *Iran. J. Ichthyol.* **7** (2), 167–180 (2020).

[27] Martínez-Abraín, A. Statistical significance and biological relevance: a call for a more cautious interpretation of results in ecology. *Acta Oecol.* **34** (1), 9–11 (2008).

[28] Hardin, G. The competitive exclusion principle: an Idea that took a century to be born has implications in ecology, economics, and genetics. *Science* **131** (3409), 1292–1297 (1960). 14399717 10.1126/science.131.3409.1292

[29] Adler, P. B., HilleRisLambers, J. & Levine, J. M. A niche for neutrality. *Ecol. Lett.* **10** (2), 95–104 (2007). 17257097 10.1111/j.1461-0248.2006.00996.x

[30] Gravel, D., Guichard, F. & Hochberg, M. E. Species coexistence in a variable world. *Ecol. Lett.* **14** (8), 828–839 (2011). 21682832 10.1111/j.1461-0248.2011.01643.x

[31] Ross, S. T. *Resource Partitioning in Fish Assemblages: a Review of Field Studies* 352–388 (Copeia, 1986).

[32] Policansky, D. The asymmetry of flounders. *Sci. Am.* **246** (5), 116–123 (1982).

[33] Fornbacke, M., Gombrii, M. & Lundberg, A. Sidedness frequencies in the flounder *Platichthys flesus* (Pleuronectiformes) along a biogeographical cline. Sarsia: North Atlantic Marine Science, **87**(5): 392–395. (2002).

[34] Das, M. & Mishra, B. On the biology of *Psettodes erumei* (Bloch & Schn.), an Indian Halibut. *Indian J. Fish.*, **37**(2): 79–92. (1990).

[35] Bowering, W. R. Age, growth, and sexual maturity of Greenland halibut, *Reinhardtius Hippoglossoides* (Walbaum). Fishery Bulletin, **81**(3): 599. (1983).

[36] HjaltiÍJákupsstovu, S. & Haug, T. Growth, sexual maturation, and spawning season of Atlantic halibut, Hippoglossus hippoglossus, in Faroese waters. *Fish. Res.* **6** (3), 201–215 (1988).

[37] Ryu, J. W. et al. Characterization of sexual size dimorphism and sex-biased genes expression profile in the Olive flounder. *Mol. Biol. Rep.* **47** (10), 8317–8324 (2020). 32981011 10.1007/s11033-020-05843-3

[38] Parker, G. A. The evolution of sexual size dimorphism in fish. *J. Fish Biol.* **41**, 1–20 (1992).

[39] Ramanathan, N. & Natarajan, R. Breeding biology of Psettodes erumei (Bloch & Schn.) and pseudorhombus Arsius (Ham. Buch.) pisces: pleuronectiformes along Porto Novo Coast. *India Aquaculture*. **18** (3), 269–282 (1979).

[40] De Roos, A. M., Boukal, D. S. & Persson, L. Evolutionary regime shifts in age and size at maturation of exploited fish stocks. *Proceedings of the Royal Society B: Biological Sciences*, **273**(1596):1873–1880. (2006). 10.1098/rspb.2006.3518 PMC1634766 16822746

[41] Hunter, A., Speirs, D. C. & Heath, M. R. *Fishery-induced Changes To Age and Length Dependent Maturation Schedules of Three Demersal Fish Species in the Firth of Clyde* **170**: 14–23 (Fisheries Research, 2015).

[42] Morgan, M. J. & Bowering, W. R. Temporal and geographic variation in maturity at length and age of Greenland halibut (Reinhardtius hippoglossoides) from the Canadian north-west Atlantic with implications for fisheries management. ICES Journal of marine Science, 54(5), pp.875–885. Munroe, T.A., 2014. Systematic diversity of the Pleuronectiformes. Flatfishes: Biology and Exploitation,13–51. (1997).

[43] Boulcott, P., Wright, P. J., Gibb, F. M., Jensen, H. & Gibb, I. M. Regional variation in maturation of sandeels in the North sea. *ICES J. Mar. Sci.* **64** (2), 369–376 (2007).

[44] Westgaard, J. I. et al. Genetic population structure in Greenland halibut (Reinhardtius hippoglossoides) and its relevance to fishery management. *Can. J. Fish. Aquat. Sci.* **74** (4), 475–485 (2017).

[45] Lin, Y. J. et al. Association To Vegetated Habitats and Different Vulnerability To Habitat Degradation for Two Fish species, Epinephelus Areolatus (Serranidae) and Siganus Canaliculatus (Siganidae), from the Western Arabian Gulf. **141**: 482–492 (Marine pollution bulletin, 2019). 10.1016/j.marpolbul.2019.03.011 30955759

[46] Froese, R. & Binohlan, C. Empirical relationships to estimate asymptotic length, length at first maturity and length at maximum yield per recruit in fishes, with a simple method to evaluate length frequency data. *J. Fish Biol.* **56** (4), 758–773 (2000).

[47] Benzik, A. N., Budanova, L. K. & Orlov, A. M. Hard life in cold waters: Size distribution and gonads show that Greenland halibut temporarily inhabit the Siberian Arctic. Water Biology and Security, **1**(2): 100037. (2022).

[48] Schilthuizen, M. & Haase, M. Disentangling true shape differences and experimenter bias: are dextral and sinistral snail shells exact mirror images? *J. Zool.* **282** (3), 191–200 (2010). 10.1111/j.1469-7998.2010.00729.x PMC3020325 21258640

[49] Adams, D., Collyer, M., Kaliontzopoulou, A. & Baken, E. Geomorph: Software for geometric morphometric analyses. R package version 4.0.10. (2025). https://cran.r-project.org/package=geomorph

[50] Core Team, R. R: A Language and Environment for Statistical Computing. R Foundation for Statistical Computing, Vienna, Austria. (2024). https://www.R-project.org/. version 4.4.2.

[51] Lin, Y. J., Qurban, M. A., Shen, K. N. & Chao, N. L. Delimitation of Tigertooth Croaker Otolithes Species (Teleostei: Sciaenidae) from the Western Arabian Gulf Using an Integrative approach, with a Description of Otolithes Arabicus sp. Nov . 58, pe10 (Zoological Studies, 2019b). 10.6620/ZS.2019.58-10 PMC6759924 31966311

[52] Claude, J. *Morphometrics with R* (Springer Science & Business Media, 2008).

[53] Jackson, C. H. Multi-State models for panel data: the Msm package for R. *J. Stat. Softw.* **38** (8), 1–29 (2011).

