# Supplementary material for: Eye-sidedness does not drive differences in growth and maturation in the Indian halibut (Psettodes erumei) from the Western Arabian Gulf
Source: Sci Rep. 2026 Jan 17;16:2203. doi: 10.1038/s41598-025-30930-5 (PMC12815957; doi:10.1038/s41598-025-30930-5)

**Appendix Figure 1.** *Psettodes erumei* in (a) dextral and (b) sinistral form. Scale bar = 5 cm.


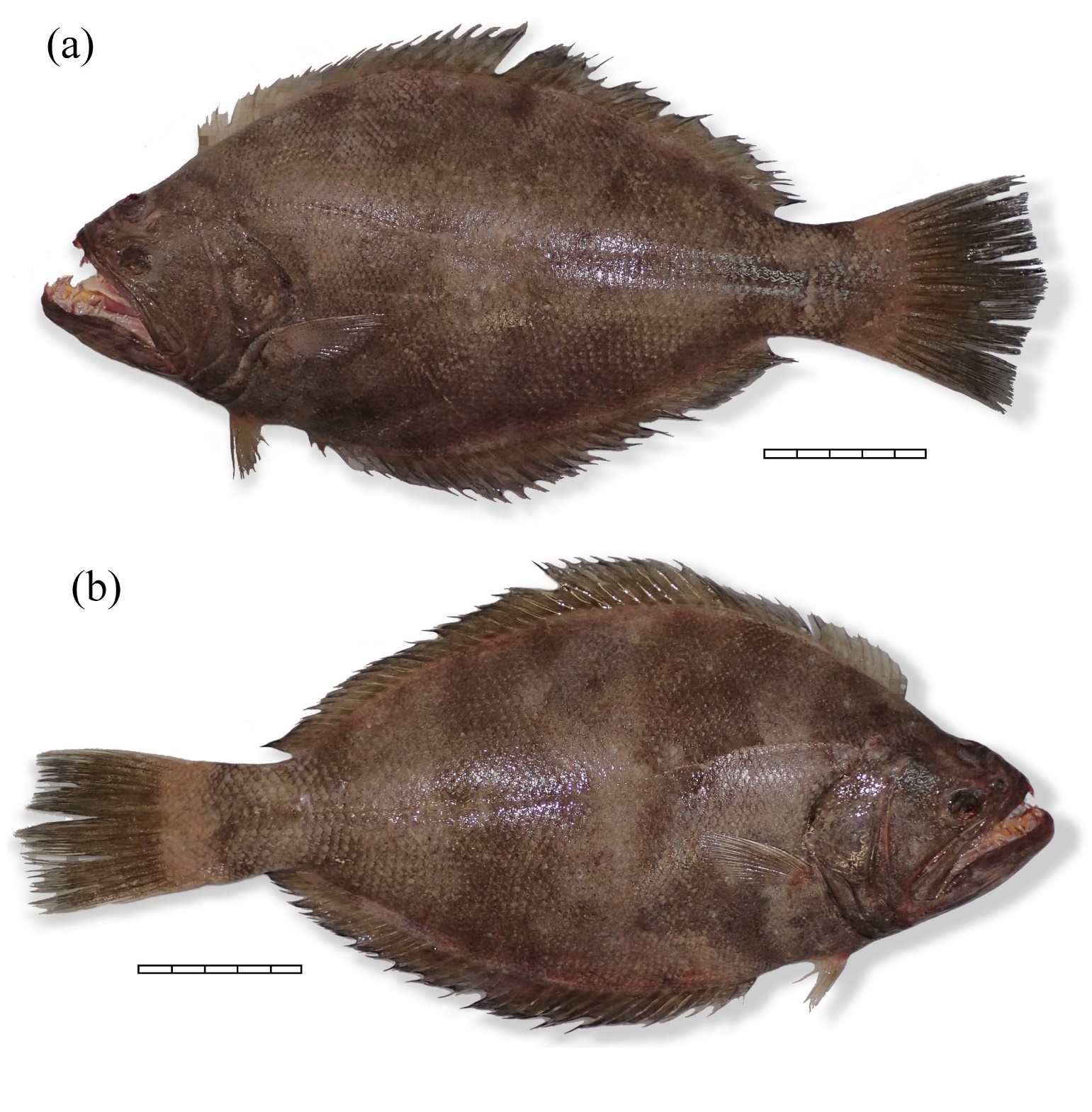


**Appendix Figure 2**. The section of the sagittal otolith of *Psettodes erumei*. Circles indicate the annuli. Scale bar = 1 mm.


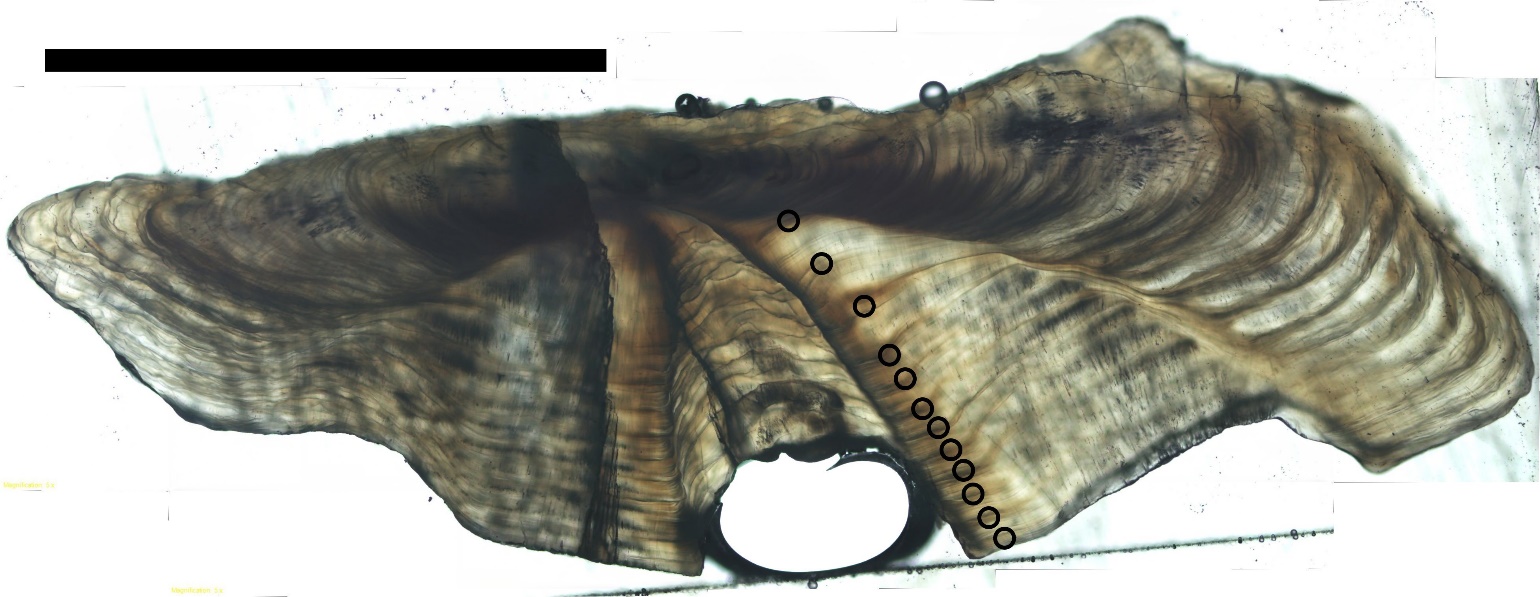

Supplement: Supplementary file 1 — Supplementary Material 1 [file 41598_2025_30930_MOESM1_ESM.docx]
